# Genome sequence of fowlpox virus from a field outbreak in Bangladesh

**DOI:** 10.1128/mra.00881-25

**Published:** 2025-11-17

**Authors:** Anandha Mozumder, Shamima Akter, Roni Mia, S. M. Nazmul Hasan, Anupam Das, Jebin Tasmin, A. B. M. Hafizur Rahman Mondal, M. Shaminur Rahman, Mohammad Imtiaj Uddin Bhuiyan, Sharmin Akter, Sukumar Saha, Md. Golzar Hossain

**Affiliations:** 1 Department of Microbiology and Hygiene, Bangladesh Agricultural University 54492, Mymensingh, Bangladesh; 2 Livestock Research Institute, Mohakhali, Dhaka, Bangladesh; 3 Bhurungamari Government College, Bhurungamari, Kurigram, Bangladesh; 4 Department of Microbiology, Jashore University of Science and Technology 421984, Jashore, Bangladesh; 5 Department of Environmental Health Science, University of Georgia 822554, Athens, Georgia, USA; 6 Department of Physiology, Bangladesh Agricultural University 54492, Mymensingh, Bangladesh; Katholieke Universiteit Leuven, Leuven, Belgium

**Keywords:** fowlpox virus, genome sequencing, Bangladesh

## Abstract

We report the complete genome sequence of a fowlpox virus (FPV) isolate from a field outbreak in Bangladesh. This genomic data will aid in comparative analyses with currently available vaccine strains and support future efforts for effective prevention and control of FPV in poultry populations.

## ANNOUNCEMENT

Fowlpox virus (FPV), a member of the *Poxviridae* family and *Avipoxvirus* genus, primarily infects chickens, pigeons, and turkeys. It causes significant economic losses in the poultry industry worldwide, including Bangladesh (1). FPV infections manifest in two clinical forms: the cutaneous (dry) form, characterized by lesions on non-feathered areas such as the legs, head, and body, and the diphtheritic (wet) form, involving lesions in the mucous membranes of the mouth and respiratory tract (2). The FPV genome consists of double-stranded DNA ranging from 260 to 309 kb pairs, making it larger than other known chordopoxvirus genomes (3). FPV has been reported in Bangladesh despite vaccination efforts. Several FPV vaccines are commercially available in the country, including Bangla Fowlpox Vac, Henpox Vet, and fowlpox vaccine. However, no complete genome sequence of FPV from field cases in Bangladesh has been available.

Nodular skin lesions were aseptically collected using sterile scissors and forceps from a live commercial layer chicken flock in Gazipur District, Bangladesh, on 18 March 2025. Viral inoculum was prepared following a previously mentioned protocol (4). A 0.2 mL viral inoculum was inoculated onto the chorioallantoic membrane of 10-day-old embryonated eggs using a sterile 1 mL tuberculin syringe with a 1/2 inch needle. The shell openings and air sacs were sealed with melted wax, and the eggs were incubated horizontally at 37°C for 5–6 days. Viral genomic DNA was extracted using the TIANamp Virus DNA/RNA Kit (Tiangen, China). Shotgun whole-genome sequencing libraries were constructed using iNextEra, a modified Illumina library preparation protocol adapted from Jones et al. (5) with adjustments to input DNA, bead-linked transposase volume, and PCR cycles to optimize yield, diversity, and genome coverage (5). DNA tagmentation was performed with Illumina bead-linked transposomes (Illumina DNA Library Prep, Illumina, Inc.), and sequencing was conducted on the Illumina NovaSeq X Plus platform (2 × 150 bp; Novogene, USA). Raw paired-end reads were quality-checked with FastQC v.0.11.9 and quality-filtered with Trimmomatic v.0.39, which retained reads longer than 50 bp. The host DNA was depleted by aligning quality-filtered reads to the *Gallus gallus* reference genome (GCA_000002315.5, GRCg6a) using BBMap (6). The remaining reads were aligned to the FPV genome (GenBank accession number NC_002188.1) using BWA-MEM v.0.7.17 (7). Sorting and indexing were performed with SAMtools v.1.12 (8), and a high-confidence consensus genome was generated using BCFtools v.1.12 (9) and VCFtools v.4.1 (10). Genome annotation was performed using Prokka v.1.14.6, and the terminal ends were determined by comparing with the previously reported complete genomes (11, 12). All software was used with default parameters.

Host DNA removal was effective, with 97.04% of reads aligning to the chicken reference genome and 2.96% retained as unmapped for downstream analysis. Subsequent alignment of host-filtered reads to the FPV reference genome produced a complete consensus sequence. A total of 25,945 reads mapped to the viral genome, covering 287,841 bases (99.76% of the reference sequence) with an average depth of ~11.9× . The mapped reads exhibited high base quality (mean base quality score 39.3) and mapping confidence (mean mapping quality 54.9), confirming robust consensus assembly. BLAST analysis revealed 99.70% nucleotide identity with previously reported FPV strains, including MH719203.1 (strain: FWPV-SD15-670.1, USA). The annotated complete genome contains 263 open reading frames, and identical inverted terminal repeats of 9.24 kbp were found at the 5′ and 3′ terminal regions, consistent with previous findings (11, 12). This study presents the complete genome sequence of an FPV isolate from Bangladesh, providing essential genomic data that may facilitate future vaccine development and aid in the control of FPV outbreaks in poultry populations.

## Data Availability

The complete genome sequence of the FPV isolate (FPV_MGH-AM01) has been deposited at GenBank, BioSample, BioProject, and SRA under the accession numbers PV982279, SAMN50183308, PRJNA1295713, and SRR34699549, respectively.
